# NEUT-SFL in Patients with COVID-ARDS: A Novel Biomarker for Thrombotic Events?

**DOI:** 10.1155/2021/4361844

**Published:** 2021-11-17

**Authors:** Laure Stiel, Yannick Rabouel, Agathe Debliquis, Valentin Pointurier, Joy Mootien, Khaldoun Kuteifan

**Affiliations:** ^1^Service de Réanimation Médicale, Groupe Hospitalier de la Région Mulhouse Sud Alsace, Mulhouse, France; ^2^Laboratoire d'Hématologie, Groupe Hospitalier de la Région Mulhouse Sud Alsace, Mulhouse, France

## Abstract

The severe acute respiratory syndrome coronavirus 2 (SARS-CoV-2) is an enveloped RNA virus first identified in December 2019 in Wuhan, China, and responsible for coronavirus disease 2019 (COVID-19). The ongoing COVID-19 pandemic is impacting healthcare worldwide. Patients who develop coagulopathy have worse outcomes. The pathophysiology of COVID-19 suggests a strong interplay between hemostasis and immune cells, especially neutrophils. Our purpose was to assess neutrophil fluorescence as a potential biomarker of deep vein thrombosis (DVT) in patients with COVID-acute respiratory distress syndrome (COVID-ARDS). Sixty-one patients with COVID-ARDS admitted to the four intensive care units (ICUs) of a French general hospital were included in this prospective study. Neutrophil activation was assessed by measuring neutrophil fluorescence (NEUT-Side Fluorescence Light, NEUT-SFL) with a specific fluorescent dye staining analyzed by a routine automated flow cytometer Sysmex XN-3000™ (Sysmex, Kobe, Japan). DVT was diagnosed by complete duplex ultrasound (CDU). We found that NEUT-SFL was elevated on admission in patients with COVID-ARDS (49.76 AU, reference value 46.40 AU, *p* < 0.001), but did not differ between patients with DVT (49.99 AU) and those without (49.52 AU, *p* = 0.555). NEUT-SFL is elevated in patients with COVID-ARDS, reflecting neutrophil activation, but cannot be used as a marker of thrombosis. Because neutrophils are at interface between immune response and hemostasis through release of neutrophil extracellular traps, monitoring their activation could be an interesting approach to improve our management of coagulopathy during COVID-ARDS. Further research is needed to better understand the pathophysiology of COVID-19 and identify high-performance biomarkers.

## 1. Introduction

The severe acute respiratory syndrome coronavirus 2 (SARS-CoV-2) is a member of the beta-coronavirus class, a family of enveloped positive-sense single-stranded RNA viruses (B). SARS-CoV-2 is responsible for the coronavirus disease 2019 (COVID-19). COVID-19 spread from Wuhan, China, where it was first identified in December 2019 and is at the origin of the ongoing COVID-19 pandemic [1, 2]. The clinical spectrum of COVID-19 ranges from asymptomatic disease to severe viral pneumonia with acute respiratory distress syndrome (ARDS) [3]. While COVID-19 typically begins as a respiratory infection, it may progress to multisystemic disease. Immune response is aimed at viral elimination and recovery. Nevertheless, overwhelming inflammatory and immune response can lead to widespread tissue damage, which in turn may be implicated in disease progression and mortality [4, 5]. This inflammatory response is associated with activation of coagulation, and indeed, a high percentage of patients with COVID-ARDS develop life-threatening thrombotic complications associated with poor prognosis [6–8]. Deep vein thrombosis (DVT) and pulmonary embolism (PE) represent the most serious thrombotic complications [7]. Arterial thrombosis, as well as clotting of catheters, dialysis membranes, or extracorporeal membrane oxygenation circuits, has also been observed [6, 9, 10]. The coagulopathy of SARS-CoV-2 infection is characterized by prominent elevation of D-dimers and fibrinogen, whereas classical coagulation biomarkers, such as prothrombin time or platelet count, remain within normal range [8, 11, 12]. This suggests uncontrolled activation of coagulation resulting in widespread microvascular thrombosis. The mechanisms of this coagulopathy are still unclear, and many molecular patterns seem to be involved, including dysregulation of the renin-angiotensin-aldosterone system (RAAS) caused by fixation of SARS-CoV-2 to angiotensin-converting enzyme 2 receptor (ACE2), oxidative stress leading to endothelial damage, and complement activation [13–15]. Most of these pathophysiological hypotheses suggest a strong interplay between immune response and hemostasis, termed immunothrombosis [16]. In this model, cytokines, platelets, immune cells, especially polymorphonuclear neutrophils (PMNs), and pathogen-associated molecular patterns induce thrombosis. Immunothrombosis participates in host defense but, if uncontrolled, can become maladaptive and induce organ dysfunction [16, 17]. The role of neutrophils in vascular cell dysfunction and aberrant host response has been studied more specifically during septic shock and septic shock-induced disseminated intravascular coagulopathy (DIC) [16, 18, 19]. Elevation of Neutrophil-Side Fluorescent Light (NEUT-SFL) was identified as a biomarker of neutrophil activation and is associated with septic shock-induced DIC [20]. During severe COVID-19, neutrophilia is predictive of severity. Pathology findings describe neutrophils in the capillaries in the lungs of autopsied COVID-19 patients [21, 22]. These observations suggest that the neutrophil could have a central role in aberrant host response inducing thrombosis in patients with COVID-ARDS.

The main objective of this study was to evaluate neutrophil fluorescence as a biomarker of deep vein thrombosis (DVT) in patients admitted for COVID-ARDS to the four intensive care units (ICUs) of the Groupe Hospitalier de la Région de Mulhouse Sud Alsace (GHRMSA).

## 2. Methods

### 2.1. Patients

Sixty-one adult patients admitted for COVID-ARDS to the four ICUs of GHRMSA were included in this study. There were no exclusion criteria. COVID-19 diagnosis was confirmed by a positive reverse-transcriptase-polymerisation chain reaction assay of a nasopharyngeal swab for SARS-CoV-2. Patients were managed according to current guidelines. Complete blood count (CBC) and complete duplex ultrasound (CDU) were performed in all patients. The Ethics Committee of our institution approved this study.

### 2.2. Demographic and Biological Characteristics

Demographic characteristics and biological parameters were collected at baseline and in the 24 hours preceding CDU exam. Simplified Acute Physiology Score (SAPS) II and Sequential Organ Failure Assessment (SOFA) score were calculated on admission.

### 2.3. DVT Definition and CDU

DVT was defined as a lower-limb vein thrombosis or as a nonleg vein thrombosis diagnosed by CDU. Only obstructive internal jugular or subclavian vein thrombosis was considered for nonleg vein thrombosis. CDU examination was performed using the General Electric vivid S6® device by two physicians of the vascular medical team of our institution.

### 2.4. Other Thrombotic Events

We did not systematically search for or record other thrombotic events. Imaging was performed if a thrombotic event was suspected based on clinical or laboratory parameters. Patients with suspected pulmonary embolism (PE) had CT pulmonary angiography (CTPA); patients with suspected arterial ischemia were evaluated by CT angiography.

### 2.5. Anticoagulation Treatment

All patients received anticoagulant treatment at admission. The choice between low molecular weight heparin (LMWH) and unfractionated heparin (UFH) treatment was determined by creatinine clearance with a 50 mL/min cut-off. Usual direct oral anticoagulants or vitamin K antagonists were substituted by LMWH or UFH. Usual antiplatelet treatment was pursued. Classic prophylactic anticoagulant therapy was defined as the administration of standard dose LMWH or UFH. Reinforced prophylactic anticoagulant therapy was defined as the administration of double dose LMWH or UFH for a targeted heparin activity between 0.2 and 0.3 IU/mL. Curative anticoagulant therapy was defined as the administration of high-dose LMWH with a targeted anti-Xa activity between 0.6 and 1.0 IU/mL or UFH with a targeted heparin activity between 0.3 and 0.5 IU/mL for patients with a history atrial fibrillation or VTE or between 0.4 and 0.6 IU/mL for patients with active VTE or mechanical cardiac valve.

### 2.6. Neutrophil Fluorescence Analysis

Blood was sampled in ethylene-diamine-tetraacetic acid (7.2 mg/4 mL) tubes on admission and on the day of the CDU. A complete blood count (CBC) was performed on a routine automated Sysmex XN-3000™ analyzer (Sysmex, Kobe, Japan) based on impedance and fluorescence flow cytometry technologies according to the manufacturer's instructions. The analyzer uses flow cytometry technology to determine the white blood cell count. Sysmex XN-3000™ is equipped with a red scatter laser light with a wavelength of 633 nm (front and side). After permeabilization by a specific Sysmex lysis reagent, three signals are recorded in the white cell differential scattergrams: forward-scattered light (FSC) (cell size), SSC (granularity), and Side Fluorescence Light (SFL) (DNA/RNA content). Neutrophil chromatin decondensation is assessed by measuring the NEUT-SFL signal generated by the incorporation of a fluorochrome-based polymethine dye that targets unpacked DNA within the permeabilized cells [23]. Binding of the dye depends on the number of available binding sites on the DNA strand; NEUT-SFL is thus a surrogate of neutrophil activation. According to the manufacturer, NEUT-SFL values in normal neutrophils are very stable [24]. The mean reference value is 46.40 arbitrary units (AU). This value has previously been validated by analyzing NEUT-SFL in more than 1300 consecutive blood samples [20]. Three control samples are processed three times daily on the analyzer and do not exceed the coefficient of variation set by the manufacturer. External controls are regularly processed according to French regulations.

### 2.7. Statistics

Quantitative variables are reported as means (range) and categorical variables as number and percentage. The normality of the distribution was assessed by the Shapiro-Wilk test. Bivariate comparisons between the DVT and no-DVT groups were performed using the Student *t*-test for quantitative parameters and the chi-square or Fisher's exact test for categorical variables. The alpha risk was set at 0.05. Statistical analysis was performed with the R software, version 4.5.2 (5R Core Team 2019, Vienna, Austria). For data presentation, asymmetrical variables are presented as median values with interquartile range using the GraphPad Prism version 8 Software (GraphPad Inc., La Jolla, CA).

## 3. Results

### 3.1. Baseline Characteristics of the Patients

Sixty-one patients with COVID-ARDS admitted to the four ICUs of GHRMSA between March 5^th^ and April 8^th^ 2020 were included in our study. Mean age was 60 (30-78) years, and 46 (75.4%) were men. Mean SAPS II was 37 (15-72), and mean SOFA score was 7 (2-14). Mean body mass index (BMI) was 32 kg/m^2^ (19.9 and 49.5), and 40 (65%) patients had BMI ≥ 30 kg/m^2^. At 28 days after admission, 15 (24.6%) patients had died. Medical history, demographic characteristics, and laboratory data are summarized in Table 1.

### 3.2. Thrombotic Events

Twenty-nine (47.5%) patients had DVT, despite prophylactic or therapeutic anticoagulant therapy since admission: 18 (29.5%) patients had lower-limb vein thrombosis, 6 (10%) patients had nonleg vein thrombosis, and five (8.1%) had both lower-limb and nonleg vein thrombosis.

CTPA was performed in twenty-one patients. Five of them had PE, of whom two had no DVT. Nine other thrombotic events were observed: seven patients who received haemodialysis presented at least one clotting of the circuit, one patient suffered multiple arterial distal necrosis, and one patient supported by veno-venous extracorporeal membrane oxygenation presented thrombotic occlusion of the centrifugal pump. In total, thirty-five (57.4%) patients developed at least one thrombotic event during their ICU stay.

### 3.3. Neutrophil Fluorescence

**Figure 1:**
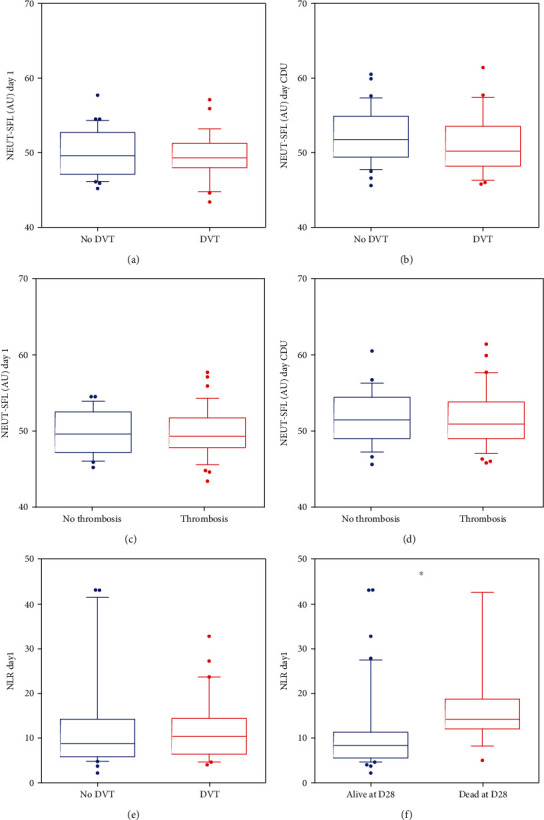
The bar inside each box represents the median; the upper and lower box limits the 25 to 75 percentiles and T-bars the 10 to 90 percentiles, respectively. (a) NEUT-SFL at day 1 in patients without DVT (blue) and with DVT (red). (b) NEUT-SFL on the day of CDU in patients without DVT (blue) and with DVT (red). (c) NEUT-SFL at day 1 in patients without thrombosis (blue) and with thrombosis (red). (d) NEUT-SFL on the day of CDU in patients without thrombosis (blue) and with thrombosis (red). (e) NLR at day 1 in patients without DVT (blue) and with DVT (red). (f) NLR at day 1 in patients who were alive at day 28 (blue) and in those who died (red).

According to the manufacturer, the NEUT-SFL mean reference value in normal neutrophils is 46.40 arbitrary units (AU) (manufacturer data). On admission, NEUT-SFL was significantly increased in patients with COVID-ARDS (49.76 AU, *p* < 0.001). Nevertheless, NEUT-SFL on admission was not significantly different between patients with DVT and those without (49.52 AU versus 49.99 AU, DVT vs. non-DVT, respectively, *p* = 0.555) (Figure 1(a)). There was no significant difference in NEUT-SFL between groups on the day of CDU (51.04 AU versus 52.34 AU, respectively, *p* = 0.178) (Figure 1(b)). The area under the ROC curve was 0.5853 (0.41L6-0.7540) (*p* = 0.3279) for DVT diagnosis at admission and 0.6000 (0.4316-0.7684) (*p* = 0.2470) on the day of the CDU. There was also no significant difference in NEUT-SFL between patients who presented any thrombotic event (49.67 AU) and those without thrombosis (49.90 AU, *p* = 0.782) (Figures 1(c) and 1(d)).

### 3.4. Leukocytes

Leukocyte and neutrophil counts were not significantly different between patients with versus without DVT at admission or on the day of CDU (Table 1). Nevertheless, an elevated neutrophil-to-lymphocyte ratio (NLR) at admission was associated with higher mortality (Figure 1(f), *p* = 0.0186) but was not associated DVT (Figure 1(e)).

## 4. Discussion

In this prospective study, NEUT-SFL was found to be elevated at admission in patients with COVID-ARDS. The values of NEUT-SFL were similar to those reported during septic shock, reflecting neutrophil activation during COVID-19 [20, 25]. In patients with septic shock, elevated NEUT-SFL was shown to be associated with DIC [20]. In our cohort, NEUT-SFL did not differ between patients with and those without DVT. Based on these observations and on the coagulation parameters previously described [6–8], the mechanisms of coagulation activation in COVID-ARDS and in septic shock-induced DIC would appear to be different. Indeed, during COVID-ARDS, fibrinogen, and D-dimers are elevated, while platelet count initially remains normal. Some patients tend to have values at the lower end of the normal range, but the severe thrombocytopenia seen in DIC is rarely observed [26]. These findings suggest that overwhelming activation of coagulation could be a local phenomenon with a “local DIC” phenotype, especially in the lungs [27]. This localized thrombosis could probably be mediated by vascular cells, especially endothelial cells and infiltrating neutrophils [21, 28]. Exploring infiltrating neutrophils, for example, in pulmonary aspirates, may be an interesting approach to improve our understanding of the disease. In this regard, although DVT is a frequent and worrisome thrombotic complication of severe COVID, it may not be representative of immunothrombosis as described in septic shock-induced DIC. Although clinical diagnosis of DVT is routinely available, macrothrombosis may not be the best way to monitor the coagulation state of severe patients with progressing disease. Nevertheless, considering COVID-induced coagulopathy as a local phenomenon remains a matter of debate [27, 29]. The multisystemic clinical features of COVID-19 suggest a systemic vascular disease with diffuse endothelial dysfunction associated with microangiopathy, with predominant lung tropism [30, 31]. Neutrophils could have an important role in this vascular disease. Indeed, neutrophilia is predictive of poor outcome in COVID patients, and elevated NLR is associated with mortality [32, 33]. We confirm this result in our cohort.

Our observations and others together suggest that enhanced neutrophil infiltration at the site of infection, associated with extensive lymphocyte depletion, may contribute to aberrant immunothrombosis during COVID-19. Elevation of NEUT-SFL indicates activation of PMNs, which promotes immune-mediated blood coagulation activation *via* different mechanisms: activated PMNs express tissue factor, leading to endothelial cell damage through degranulation and production of reactive oxygen species [34]. Activated PMNs also release microparticles and neutrophil extracellular traps (NETs) [16, 18, 35]. Furthermore, they interact with platelets, forming activated platelet-neutrophil complexes that trigger an immune response to infection through neutrophil recruitment and NET production [36]. Barnes et al. reported a histological description of lungs from 3 necropsies of patients with COVID-ARDS in April 2020 [21]. They described interstitial neutrophilic infiltration with small vessel occlusions containing PMNs and neutrophil fragments consistent with NETs [21, 37]. It remains uncertain whether these modifications are specific to SARS-CoV-2 infection or a final common stage in the thromboinflammatory response to fatal viral infections [14, 28]. Neutrophil infiltration is probably mediated by downregulation of ACE2 expressed by pulmonary epithelial cells on SARS-CoV-2 infection [38, 39]. Although the detection of NETs remains controversial, and the evidence in COVID-ARDS is limited (albeit growing), PMNs seem to play an important role in the pathophysiology of complications of COVID-19. Underlying comorbidities like diabetes or cardiovascular diseases, associated with a risk of severe forms of COVID-19, also contribute to persistent exaggerated neutrophil activation [38].

Altogether, NEUT-SFL does not seem to be a good marker of DVT during COVID-19. Perhaps it is not specific enough, because it reflects the different pathways of PMN activation (e.g., phagocytosis, NETosis and degranulation, to mention but a few) [19, 40]. Biomarkers of NETs could be more representative of the role of PMNs in COVID hypercoagulability, as previously reported in other forms of ARDS [41]. Recent postmortem findings confirm the presence of NETs in the lungs of patients who died from COVID-19 [42]. Elevation of two indirect markers of NETosis, namely, cell-free DNA and MPO-DNA, in the plasma of patients with severe forms of COVID-19 has also been reported [43]. Nevertheless, because of the ongoing COVID-19 pandemic, it is of paramount importance to identify routinely available biomarkers that could improve physician decision-making for treatment or orientation of patients. Our preliminarily observational study has some limitations and was not designed to demonstrate the pathophysiology of thrombosis during COVID-ARDS. Further studies exploring more haematological markers in a large panel of patients could help to guide clinical decision-making at the bedside, pending a more comprehensive approach to the disease based on translational research.

## 5. Conclusion

To the best of our knowledge, we report the first cohort to investigate the association between neutrophil activation, as measured by NEUT-SFL, and DVT in patients with COVID-ARDS. Our data highlights NEUT-SFL as a marker of neutrophil activation during COVID-ARDS, but not as a marker of DVT or other macrothrombosis. NEUT-SFL cannot be used as a marker of thrombosis in COVID-19 patients. COVID-ARDS represents a state of overwhelming inflammatory response to infection associated with hypercoagulation, which induces multiorgan failure. For now, lymphocyte count, NLR, and D-dimers offer the better prognostic information in the management of the disease, but they are not sufficient. Therefore, new biomarkers measuring vascular cell activation are needed to monitor the state of the disease and perhaps enable case-by-case management. Neutrophils nonetheless remain an interesting target for further research, because they are at interface between immune response and hemostasis. Further translational research is needed to better understand the pathophysiology of COVID-19.

## Figures and Tables

**Table 1:** Characteristics of the patients.

Characteristics	All patients (*n* = 61)	No DVT (*n* = 32)	DVT (*n* = 29)	*p* value
Demographic parameters				
Sex, no. (%)				
Male	46 (75.4)	24 (75.0)	22 (75.9)	1.000
Age—years, mean (sd)	60.44 (11.43)	58.88 (11.56)	62.17 (11.23)	0.264
BMI—kg/m^2^, mean (sd)	32.04 (5.85)	31.82 (6.48)	32.29 (5.18)	0.759

Severity of the disease				
SAPS II, mean (sd)	37.33 (11.62)	38.62 (12.69)	35.90 (10.34)	0.193
SOFA, mean (sd)	6.7 (2.7)	7.23 (2.78)	6.31 (2.59)	0.193
Death at 28 days, no. (%)	15 (24.6)	7 (21.9)	8 (27.6)	1.000

Days between ICU admission and CDU, mean (sd)	9.14 (6.13)	8.53 (6.14)	10.34 (6.11)	0.253

Medical history, no. (%)				
Arterial hypertension	28 (45.9)	12 (37.5)	16 (55.2)	0.260
Diabetes mellitus	23 (37.7)	12 (37.5)	11 (37.9)	1.000
Dyslipidaemia	18 (29.5)	9 (28.1)	9 (31.0)	1.000
Coronary artery disease	11 (18.0)	6 (18.8)	5 (17.2)	1.000
Cancer	7 (11.5)	3 (9.4)	4 (13.8)	0.890
Active smoking	5 (8.2)	5 (15.6)	0 (0.0)	0.064
COPD	8 (13.1)	5 (15.6)	3 (10.3)	0.818
Asthma	7 (11.5)	1 (3.1)	6 (20.7)	0.081
OSAS	7 (11.5)	2 (6.2)	5 (17.2)	0.346

Medication, no. (%)				
ACEI	10 (16.4)	3 (9.4)	7 (24.1)	0.227
ARB	12 (19.7)	7 (21.9)	5 (17.2)	0.895
Antiplatelet aggregation drugs	14 (23.0)	8 (25.0)	6 (20.7)	0.924
Long-term anticoagulant treatment	5 (8.2)	2 (6.2)	3 (10.3)	0.920

Haematological parameters at admission				
Leukocyte count—109/L, mean (sd)	9.23 (4.83)	9.65 (5.71)	8.76 (3.63)	0.485
Lymphocyte count—109/L, mean (sd)	0.83 (0.38)	0.80 (0.33)	0.86 (0.42)	0.737
Neutrophil count—109/L, mean (sd)	7.31 (3,86)	7.19 (4,53)	7.45 (2.89)	0.048
Platelet count—109/L, mean (sd)	254 (111)	258 (111)	248 (112)	0.507
D-dimers—mg/L, mean (sd)	3.42 (5.33)	2.03 (3.11)	4.81 (6.67)	0.091
PT activity %; mean (sd)	72 (13.7)	70 (9.5)	73 (17.2)	0.469

Haematological parameters on day of CDU				
Leukocyte count—109/L, mean (sd)	11.8 (4.18)	11.5 (3.81)	12 (4.61)	0.683
Lymphocyte count—109/L, mean (sd)	1.17 (0.51)	1.30 (0.55)	1.04 (0.44)	0.049
Neutrophil count—109/L, mean (sd)	9.16 (3,65)	8.70 (3,17)	9.70 (3.85)	0.361
Platelet count—109/L, mean (sd)	344 (144)	330 (153)	359 (135)	0.438
D-dimers—mg/L, mean (sd)	4.45 (4.27)	3.12 (2.02)	5.90 (5.52)	0.029
PT activity %; mean (sd)	70 (8.8)	68 (8.1)	73 (8.9)	0.034

Neutrophil fluorescence				
NEUT-SFL day 1—AU (sd)	49.76 (3,07)	49.99 (3.42)	49.52 (3.16)	0.555
NEUT-SFL day CDU—AU (sd)	51.73 (3,72)	52.34 (3.43)	51.04 (3.55)	0.178

BMI: body mass index; CDU: complete duplex ultrasound; COPD: chronic obstructive pulmonary disease; NEUT-SFL: Neutrophil-Side Fluorescent Light; OSAS: obstructive sleep apnoea syndrome; PT: prothrombin time; SAPS II: Simplified Acute Physiology Score II; SOFA: Sequential Organ Failure Assessment; DVT: deep vein thrombosis.

## Data Availability

Data are available on request.
